# Supplementary material for: Causal Relationship between Aging and Anorexia Nervosa: A White-Matter-Microstructure-Mediated Mendelian Randomization Analysis
Source: Biomedicines. 2024 Aug 16;12(8):1874. doi: 10.3390/biomedicines12081874 (PMC11351342; doi:10.3390/biomedicines12081874)
Supplement: Supplementary file 1 [file biomedicines-12-01874-s001.zip › Supplementary Figure/Figure S4/Singe SNP plot of MR analysis for TL to ubm-b-1792.pdf]

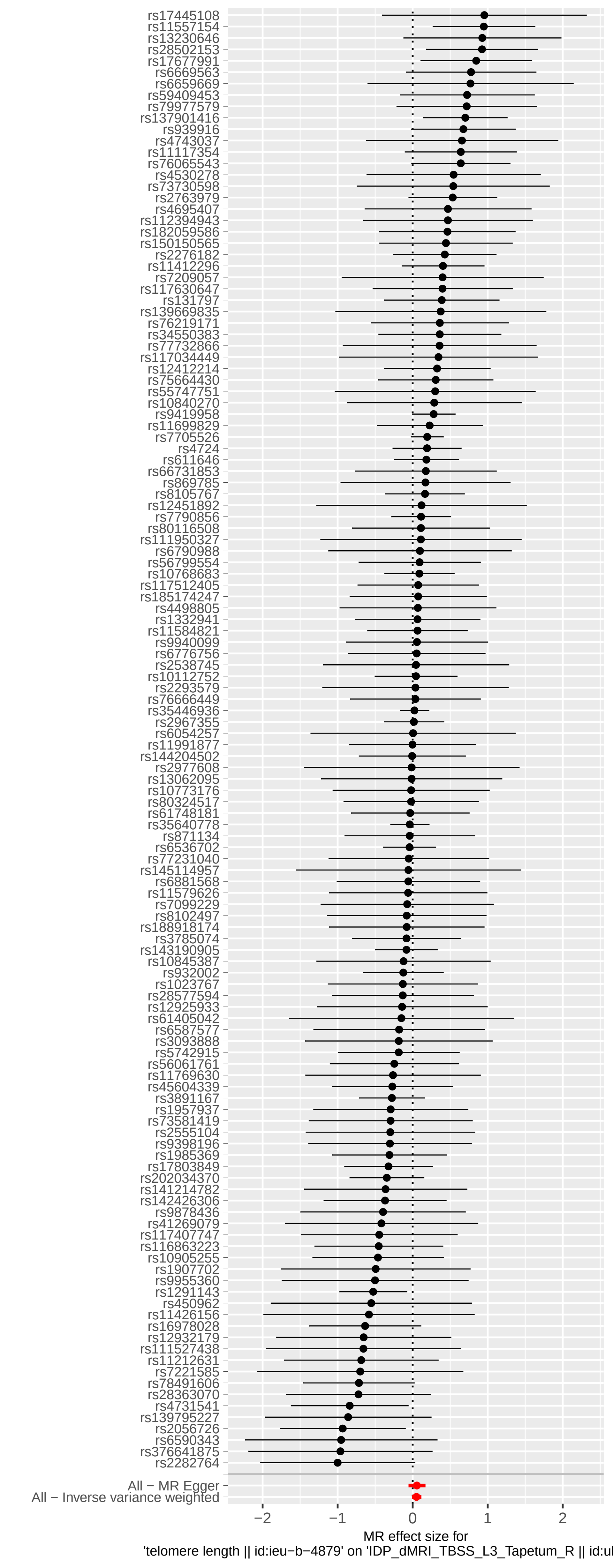

rs17445108
rs11557154
rs13230646
rs28502153
rs17677991
rs6669563
rs6659669
rs59409453
rs79977579
rs137901416
rs939916
rs4743037
rs11117354
rs76065543
rs4530278
rs73730598
rs2763979
rs4695407
rs112394943
rs182059586
rs150150565
rs2276182
rs11412296
rs7209057
rs117630647
rs131797
rs139669835
rs76219171
rs34550383
rs77732866
rs117034449
rs12412214
rs75664430
rs55747751
rs10840270
rs9419958
rs11699829
rs7705526
rs4724
rs611646
rs66731853
rs869785
rs8105767
rs12451892
rs7790856
rs80116508
rs111950327
rs6790988
rs56799554
rs10768683
rs117512405
rs185174247
rs4498805
rs1332941
rs11584821
rs9940099
rs6776756
rs2538745
rs10112752
rs2293579
rs76666449
rs35446936
rs2967355
rs6054257
rs11991877
rs144204502
rs2977608
rs13062095
rs10773176
rs80324517
rs61748181
rs35640778
rs871134
rs6536702
rs77231040
rs145114957
rs6881568
rs11579626
rs7099229
rs8102497
rs188918174
rs3785074
rs143190905
rs10845387
rs932002
rs1023767
rs28577594
rs12925933
rs61405042
rs6587577
rs3093888
rs5742915
rs56061761
rs11769630
rs45604339
rs3891167
rs1957937
rs73581419
rs2555104
rs9398196
rs1985369
rs17803849
rs202034370
rs141214782
rs142426306
rs9878436
rs41269079
rs117407747
rs116863223
rs10905255
rs1907702
rs9955360
rs1291143
rs450962
rs11426156
rs16978028
rs12932179
rs111527438
rs11212631
rs7221585
rs78491606
rs28363070
rs4731541
rs139795227
rs2056726
rs6590343
rs376641875
rs2282764
All − MR Egger
All − Inverse variance weighted
−2
−1
0
1
2
MR effect size for
'telomere length || id:ieu−b−4879' on 'IDP_dMRI_TBSS_L3_Tapetum_R || id:ul
